# Colorectal Carcinomas: Searching for New Histological Parameters Associated with Lymph Node Metastases

**DOI:** 10.3390/medicina59101761

**Published:** 2023-10-02

**Authors:** Aura Jurescu, Adrian Văduva, Octavia Vița, Adelina Gheju, Remus Cornea, Codruța Lăzureanu, Anca Mureșan, Marioara Cornianu, Sorina Tăban, Alis Dema

**Affiliations:** 1 Department of Microscopic Morphology-Morphopathology, ANAPATMOL Research Center, “Victor Babeş” University of Medicine and Pharmacy, 300041 Timișoara, Romania; 2 Department of Pathology, “Pius Brînzeu” County Clinical Emergency Hospital, 300723 Timişoara, Romania; 3 Emergency County Hospital Deva, 330032 Deva, Romania

**Keywords:** colorectal carcinomas, prognostic factors, tumor budding, poorly differentiated clusters, lymph node metastasis, tumor border configuration

## Abstract

*Background and Objectives*: Colorectal cancer (CRC) continues to be an essential public health problem. Our study aimed to evaluate the prognostic significance of classic prognostic factors and some less-studied histopathological parameters in CRC. *Materials and Methods*: We performed a retrospective study on 71 colorectal carcinoma patients who underwent surgery at the “Pius Brînzeu” County Clinical Emergency Hospital in Timișoara, Romania. We analyzed the classic parameters but also tumor budding (TB), poorly differentiated clusters (PDCs) of cells, tumor-infiltrating lymphocytes (TILs), and the configuration of the tumor border on hematoxylin–eosin slides. *Results*: A high degree of malignancy (*p* = 0.006), deep invasion of the intestinal wall (*p* = 0.003), an advanced stage of the disease (*p* < 0.0001), lymphovascular invasion (*p* < 0.0001), perineural invasion (*p* < 0.0001), high-grade TB (*p* < 0.0001), high-grade PDCs (*p* < 0.0001), infiltrative tumor border configuration (*p* < 0.0001) showed a positive correlation with lymph node metastases. *Conclusions*: The analyzed parameters positively correlate with unfavorable prognostic factors in CRC. We highlight the value of classic prognostic factors along with a series of less-known parameters that are more accessible and easier to evaluate using standard staining techniques and that could predict the risk of relapse or aggressive evolution in patients with CRC.

## 1. Introduction

Colorectal cancer (CRC) is one of the most common malignancies worldwide and is first among gastrointestinal cancers [1]. Although in the last decades, significant progress was made regarding the early detection, identification of prognostic markers, and therapeutic management of this cancer [2], elevated morbidity and mortality rates for CRC are still recorded.

TNM stage (tumor, node, metastasis), established according to the system recommended by the American Joint Committee on Cancer/the Union for International Cancer Control (AJCC/UICC), represents the most important prognostic factor for CRC and guides therapeutic options in clinical practice [1]. However, the actual value of this parameter is questionable since a significant number of patients with stage III CRC have a favorable evolution. In contrast, other patients with stage I/II show more aggressive disease progress [3]. It has been shown that approximately 10–25% of patients with early-stage CRC had an unfavorable evolution [4]. Consequently, the current TNM AJCC classification of CRC offers limited prognostic information and does not consider patients’ responses to therapy [5]. Thus, parameters that could predict the occurrence of lymphatic and distant metastases in early-stage CRC patients could guide clinicians in therapeutic management [6]. Risk stratification models based on clinical–morphological parameters have been studied but proved insufficient, as a significant number of patients classified as high-risk underwent unnecessary surgery [7]. Therefore, an intense concern regarding the identification, validation, and implementation of new parameters can be observed globally irrespective of the disease stage that can anticipate CRC patients' progress and predict their response to therapy. In light of the presented data, we developed this study starting from the premise that by the complex and complete histopathological interpretation of surgical pieces, new parameters can be identified to predict the risk of the aggressive evolution of the disease.

Although the analysis of certain molecular factors seems valuable and attractive when establishing the prognostic and predictive signification for patients with CRC [1,8], a series of parameters that can be interpreted on slides with the usual hematoxylin–eosin (HE) and immunohistochemical (IHC) stains are of interest lately, making them easier to assess and more accessible than molecular markers. Some of these new parameters, like tumor budding (TB) [9,10], poorly differentiated clusters (PDCs) of cells [11,12], the configuration of the invasion front/tumor border configuration [13], and tumor-infiltrating lymphocytes (TILs) [14] could complete or even replace some of the traditional prognostic factors in CRC.

In this context, the present study aimed to evaluate some modern histopathological parameters interpreted on HE slides and to perform a comparative analysis of the newly assessed parameters with the traditional prognostic factors to establish their prognostic significance. Thus, we assessed a series of classical prognostic factors for CRC. In addition, we evaluated several modern parameters, such as TB, PDCs, TILs, and the tumor border configuration, that still need a standardized method of assessment and validation to be included in the CRC histopathological report.

## 2. Materials and Methods

### 2.1. Study Design and Participants

We developed a retrospective study on a group of 71 cases of CRC, including 50 consecutive cases of colorectal carcinomas, diagnosed and treated by classical surgical intervention in 2014 and 21 consecutive cases of colorectal carcinomas robotically operated (da Vinci Xi^®^ Surgical System) at the Surgery Clinic II of the County Clinical Emergency Hospital “Pius Brȋnzeu” Timişoara (CCEHPBT) between July 2015 and July 2016. Our study included the cases of colorectal carcinomas diagnosed on resection specimens. Criteria for exclusion from the study lot: patients with colorectal carcinomas diagnosed on biopsies; patients with different types of cancer but carcinomas; patients who received neoadjuvant therapy before the surgical treatment; and patients with tumor recurrences. CRC patients treated with chemotherapy and/or radiotherapy before surgery were excluded because of significant changes in the macroscopic and microscopic appearance of the tumor. After the neoadjuvant therapy, the initial morphology of the tumor is subject to substantial alterations, and the evaluation of histopathological parameters suffers certain limitations, especially in tumors with broad fibrotic areas, for example, in cases with a major therapeutic response.

### 2.2. Data Sources and Variables of Interest

Clinical and morphological data were collected from the Pathology Department database of our hospital. We analyzed the accompanying sheets of the biopsy material, the clinical observation sheets of the patients, and the histopathological results. After we selected the cases to be included in the study, the tumor sections stained with HE were reevaluated to complete the histopathological data needed. For each case, we selected a representative slide containing the most profound tumor infiltration into the intestinal wall, including the front of tumor invasion. These slides were allocated for evaluating new parameters to two junior pathologists for double-blind verification, and discordant results were clarified by a senior pathologist.

Therefore, the following parameters were entered into an Excel table-type database and analyzed: sex, age, localization of the tumors, histological type of the tumors, degree of tumor differentiation (G) according to the World Health Organization (WHO grade), tumor grade according to TB (GBd), tumor grade according to PDCs (PDCs-G), depth of tumor invasion (pT), lymph node status (pN), pathologically documented distant metastases (pM1), AJCC stage of the disease (according to the TNM AJCC system), lymphatic and vascular invasion (LVI), perineural invasion (PNI), tumor necrosis, tumor ulceration, TILs, and configuration of the tumor invasion front.

### 2.3. Demographic Data and Histopathological Parameters Analysis

#### 2.3.1. Patients’ Age and Tumor Location

The patients were assigned to the following age groups: 41–50, 51–60, 61–70, 71–80, and 81–90 years old. To make the statistical analysis more accessible, we divided the patients into two groups according to their age: patients < 65 years old and patients ≥ 65 years of age, respectively. According to their localization, the tumors were grouped as follows: tumors of the right colon—tumors developed in the cecum, ascending colon, hepatic angle of the colon, and transverse colon; tumors of the left colon—tumors of the splenic angle, descending colon, sigmoid colon, and rectal tumors (including the tumors of the rectal-sigmoid junction).

#### 2.3.2. Histological Subtype and Degree of Differentiation of Tumors

The histological subtype and the differentiation degree of the tumors were assigned according to WHO classification [15]. Regarding the histological subtype, CRC can be divided into conventional adenocarcinomas (ADKs) (the usual type or NOS—not otherwise specified) or particular types of carcinomas. Neoplasms with extracellular lakes of mucin representing >50% of the tumor area in which tumor cells can be identified were considered mucinous carcinomas. In comparison, neoplasms with a mucinous component representing <50% of the tumor were defined as ADKs with a mucinous contingent [15]. ADKs with a mucinous contingent were assessed with the other NOS/conventional ADKs.

The histological differentiation degree of CRC was evaluated for ADK NOS using three grading systems: the usual grading system—WHO grade and the grading system based on TB (GBd) and PDCs (PDCs-G) quantification, respectively. Because there is no consensus regarding the definition of TB and PDCs in the presence of mucin, we did not grade the cases of mucinous ADKs with any of the three grading systems.

- Evaluation of the WHO grade

The WHO grade was assessed for ADK NOS based on the percentage of gland formation as follows: G1, G2, G3—glandular structure formation in >95%, 50–95%, 0–49% of the tumor area, respectively, and G4—no gland formation or other mucinous differentiation, squamous, or neuroendocrine features [15]. Moreover, we divided the tumors into two groups of differentiation degrees depending on the percentage of gland formation (≥50% and <50%): tumors with a low degree of malignity (G1–G2) and high degree malignant tumors (G3–G4) according to WHO recommendations [15,16,17].

- Evaluation of GBd grade

Regarding TB, we graded ADK NOS on HE-stained sections using an optical microscope quantifying the presence of TB on the invasion front of the tumor according to the recommendations proposed at the International Tumor Budding Consensus Conference (ITBCC) in 2016 [18]. TB was defined as isolated cells or groups of ≤4 tumor cells that were not glandular areas fragmented by the inflammation [19]. To quantify TB, we reevaluated each case and selected the slides that included the invasion front from the area with the maximum infiltration into the intestinal wall. The tumor invasion front was examined under low magnification (10×) in 10 microscopical fields, and the field with the highest density of TB (hotspot) was selected for evaluation; the latter was then examined under intermediary magnification (200×, field area of 0.785 mm^2^). After that, CCR cases were divided into three categories according to GBd: G1Bd, G2Bd, and G3Bd—tumors with 0–4 TB, 5–9 TB, and ≥10 TB, respectively, according to the recommendations of the ITBCC in 2016 [18].

- Evaluation of PDCs-G grade

Also, we graded ADK NOS depending on the presence of PDCs at the tumor invasion front on HE-stained slides according to the method introduced by Ueno et al. in 2012 [20]. Initially, the slides were examined under an optical microscope at low magnification (40×) to identify the area with the highest density of PDCs along the invasion front (hotspot) that was then evaluated at an intermediary magnification (200×, field area of 0.785 mm^2^). Depending on the number of PDCs identified in the analyzed area, we classified the CRC cases into three categories according to the method introduced by Ueno et al. in 2012 [20] and also used by Barresi et al. [21]. The quantification system for PDCs is similar to that of TB: tumors with <5 PDCs were considered PDCs-G1, those with 5–9 PDCs were appointed to the PDCs-G2 category, and cases with ≥10 PDCs were considered PDCs-G3.

#### 2.3.3. TNM Stage, Necrosis, Ulceration, Lymphovascular, and Perineural Invasion

The pTNM parameters were established using the staging protocol proposed by the AJCC Staging Manual [22], and the cases were classified accordingly. Additionally, the cases were grouped into CRC with incipient invasion (pT1–pT2) and tumors with profound invasion (pT3–pT4) into the intestinal wall. We assessed tumor necrosis as reduced/absent (<10% of the tumor mass) or present (≥10% of the tumor mass). Parameters such as the presence of ulceration on the tumor surface, lymphatic and vascular invasion (LVI), perineural invasion (PNI), and the status of regional lymph nodes (pN) were quantified as absent/present.

#### 2.3.4. Assessment of TILs and Tumor Border Configuration

We assessed the absence/presence of TILs in the tumor invasion front. We classified the cases with a binary system: TILs- for the cases with no inflammatory cells or a quantity of ≤5% on a field at 400× magnification and TILs+ for cases with a high number of lymphocytes in the tumor invasion front, sometimes presenting as a band, affecting the adjacent groups of tumor cells according to the model described by Schwarz et al. [23]. We considered TILs as mononucleate cells (lymphocytes and plasma cells) and did not quantify granulocytes or macrophages. The configuration of the tumor invasion front was regarded as the “pushing” type when we observed a linear/continuous model of tumor expansion or the “infiltrating” type when an irregular invasion model was present. For the cases presenting both characteristics of the invasion front, we considered the more prominent model and classified them accordingly.

Two articles partially described this group of patients regarding socio-demographic characteristics and clinical –pathological parameters [24,25]. In the present study, we performed a multivariate analysis between the morphological parameters studied to identify the relationship between them and to establish the prognostic significance of the classical and less studied parameters, such as TILs and the configuration of the invasion front/tumor invasion model. The multivariate analysis was performed only for the cases of ADK NOS (except for the two cases of mucinous ADK).

### 2.4. Statistical Analysis

The parameters we gathered were statistically analyzed using the GraphPad Prism software, v8.2 (GraphPad Software Inc., San Diego, CA, USA) and IBM SPSS v25 (IBM Corporation Armonk, NY, USA) software. We used the Pearson correlation coefficient (r) to highlight the associations between the analyzed data. The “*r*” value is statistically significant if it approaches 1; the closer “*r*” is to 1 in absolute value, the stronger the correlation. The interpretation of “*r*” was carried out as follows: 1.0—perfect association; 0.8 to 1.0—very strong association; 0.6 to 0.8—strong association; 0.4 to 0.6—moderate association; 0.2 to 0.4—weak association; 0.0 to 0.2—very weak or no association. The “*p*” value was considered statistically significant if it was lower than 0.05.

## 3. Results

### 3.1. Histopathology and Patient Characteristics

We identified 71 cases that met the inclusion criteria of our study. The demographic and clinical–pathological data of the patient group are presented in Table 1.

**Figure 1:**
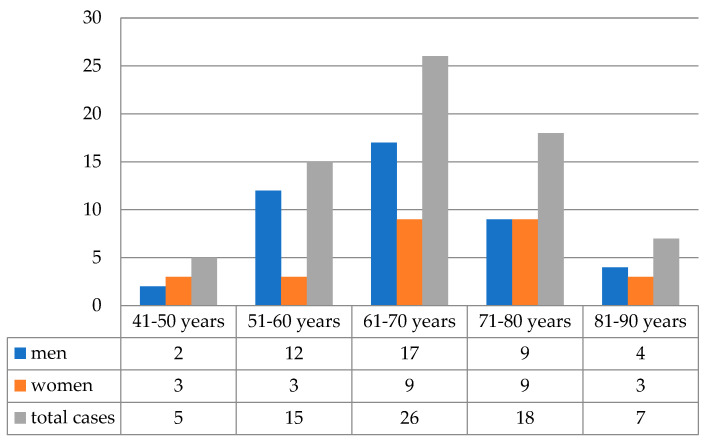
The distribution of cases according to age groups (*n* = 71).

The studied group of patients comprised 44 (62.0%) men and 27 (38.0%) women with a mean age of 66.47 years old at the time of diagnosis. Of the 71 patients, 43 (60.6%) were ≥65 years old. The highest number of cases was identified in men’s seventh decade of life—17/44 patients (38.6%). In women, the highest incidence was encountered in the seventh and eighth decades of life with a similar percentage of cases—9/27 (33.5%), as seen in Figure 1.

**Figure 2:**
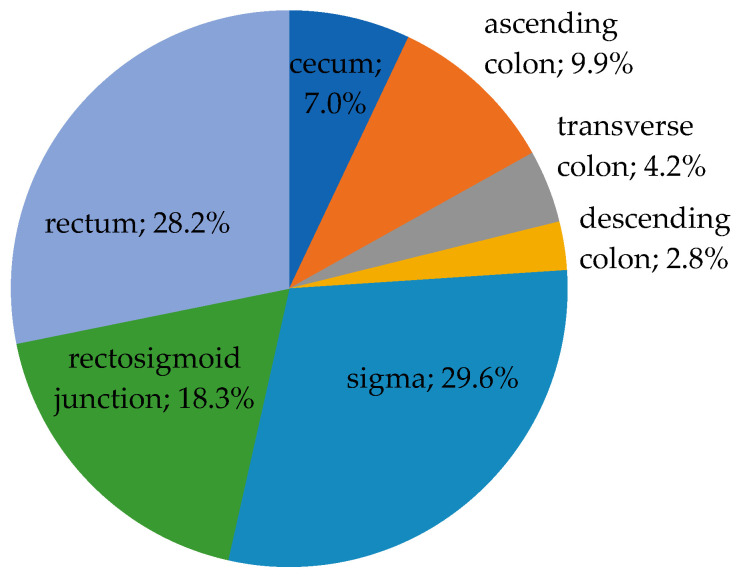
The distribution of cases according to tumor location (*n* = 71).

The tumors were localized in the following regions: cecum—5 cases (7.0%), ascending colon—7 cases (9.9%), transverse colon—3 cases (4.2%), descending colon—2 cases (2.8%), sigmoid colon—21 cases (29.6%), rectosigmoid junction—13 cases (18.3%), and rectum in 20 cases (28.2%). According to our criteria of classification for the localization of the tumors, in 15 cases (21.1%), the tumors were found in the right colon, 23 cases (32.4%) in the left colon, and 33 cases (46.5%) were identified in the rectum, as seen in Figure 2.

Regarding the histological type of the tumors, 69 were conventional/NOS ADKs, of which, 16 (22.5%) presented a mucinous component, and 2 cases (2.8%) were mucinous ADKs. Because of the low number of mucinous ADKs, we did not perform the statistical analysis according to the histological type.

**Figure 3:**
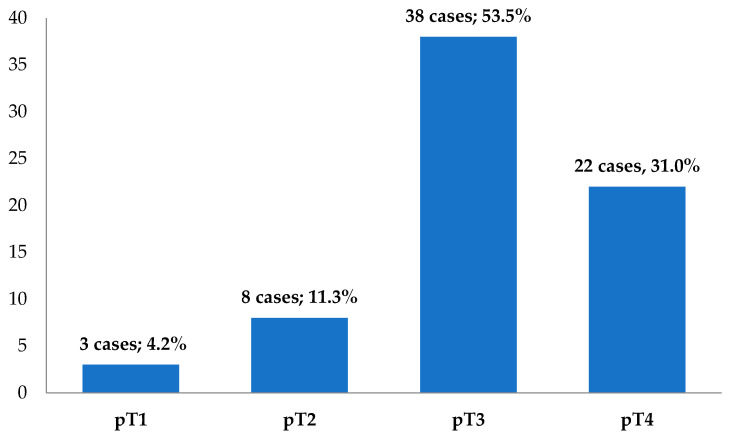
The distribution of cases according to the pT parameter (*n* = 71).

The cases were divided according to the depth of tumor invasion into the intestinal wall (pT), as seen in Figure 3. Using the binary classification, 11 cases (15.5%) were tumors with incipient invasion (pT1–pT2), but the majority of cases—60 (84.5%)—presented a deep invasion into the intestinal wall (pT3–pT4).

**Figure 4:**
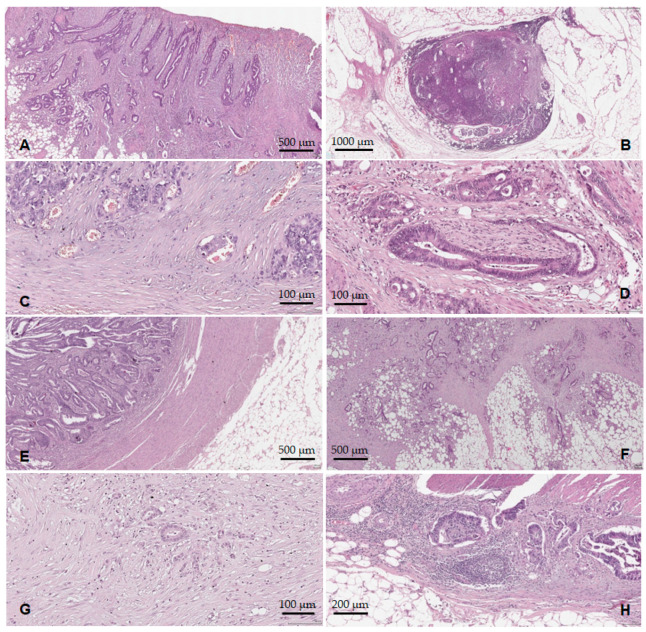
Histopathological features of the ADK NOS cases: (**A**) tumor surface ulceration; (**B**) lymph node metastases; (**C**) lymphovascular invasion; (**D**) perineural invasion; (**E**) tumor border configuration—the pushing type; (**F**) tumor border configuration—the infiltrative type; (**G**) TILs reduced/absent; (**H**) TILs present.

Tumor ulceration (Figure 4A) was identified in 61 cases (85.9%), and tumor necrosis in 47 cases (66.2%). Concerning the lymph node status, 34 cases (47.9%) showed metastases into the regional lymph nodes (pN+), Figure 4B. In six cases (8.4%), distant metastases were documented (pM1). Lymphovascular invasion (LVI+) was identified in 28 cases (39.4%), Figure 4C. We should note that all 28 LVI+ cases were pT3–pT4, pN+. PNI was present in 23 cases (32.4%), all from the pT3–pT4 group. From the PNI+ cases (Figure 4D), 19 (82.6%) were also LVI+ and pN+. Regarding the configuration of the tumor invasion front (pushing vs. infiltrative), 26 cases (36.6%) presented the pushing type (Figure 4E), while 45 cases (63.4%) had an infiltrative type of invasion (Figure 4F). We noted that 27 of the LVI+ cases (96.4%) presented an infiltrative configuration of the tumor invasion front. Considering the absence/presence of the lymphocytic infiltrate in the tumor invasion front, 10 cases (15.5%) were classified as TIL− (Figure 4G), while 60 cases (84.5%) showed TILs+ (Figure 4H).

### 3.2. Comparison Amongst the Three Grading Systems

**Figure 5:**
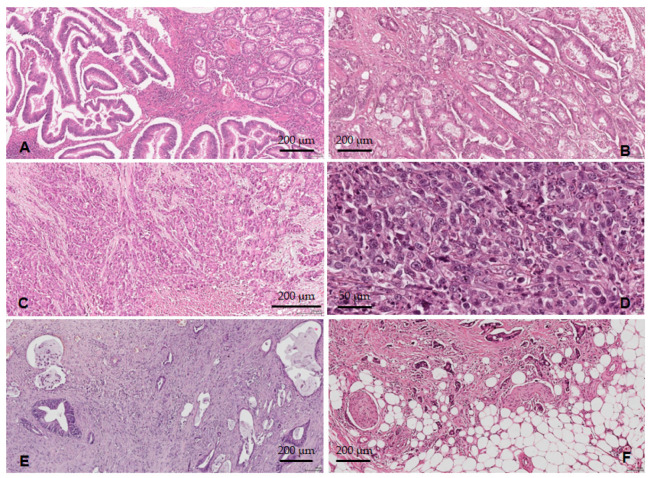
The histological differentiation degree: (**A**) ADK NOS G1—well-differentiated tumors; (**B**) ADK NOS G2—moderately differentiated tumors; (**C**) ADK NOS G3—poorly differentiated tumors; (**D**) ADK NOS G4—undifferentiated carcinomas; (**E**) low-grade TB and PDCs; (**F**) high-grade TB and PDCs.

The histological differentiation degree of CRC was evaluated using three grading systems: WHO grade, GBd, and PDCs-G only for the 69 cases of ADK NOS, as shown in Table 2, Figure 5.

With the WHO grade, we observed that 8.7% of cases were considered G1 (Figure 5A), and the majority (79.7%) were G2 (Figure 5B) followed by 11.6% G3–G4 (Figure 5C,D). Using the GBd grading system, we observed the following distribution of cases: 23.2% G1Bd, 27.5% G2Bd, and 49.3% G3Bd. According to the PDCs-G grading system, 16% of cases were PDCs-G1, 44.9% PDCs-G2, and 39.1% PDCs-G3 (Figure 5E,F).

### 3.3. The Results of the Multivariate Analysis

In addition, we performed multivariate analyses using the Pearson correlation coefficient for the 69 cases of ADK NOS, see Table S1 in Supplementary Materials.

Neither the sex of the patients nor their age correlated significantly with any other prognostic factor we analyzed. Regarding topography, we observed a weak negative correlation between the localization of the tumors (right colon/left colon/rectum) and their extension into the intestinal wall (pT1–pT2/pT3–pT4), r = −0.270, *p* = 0.025. Thus, rectal tumors in stages pT1–pT2 were found in 25.0% of cases (*n* = 5/20) compared to tumors of the right colon diagnosed in pT3–pT4 stages in 100% of cases. Also, a weak negative relationship was observed between tumor localization (right/left colon, rectum) and the type of the invasion front (pushing/infiltrative), r = −0.253, *p* = 0.036. In 40.0% of cases (*n* = 8/20), rectal tumors presented a pushing type of invasion front in contrast to right colon tumors, which showed this invasion model in 23.08% of cases (*n* = 3/13).

The multivariate analyses we carried out revealed positive correlations between the WHO grade and the following parameters: GBd (*p* = 0.005), PDCs-G (*p* < 0.0001), pT (*p* < 0.0001), pN (*p* = 0.006), TNM AJCC stage (*p* = 0.001), LVI (*p* = 0.004), PNI (*p* = 0.013), and the configuration of the invasion front (*p* = 0.001). We noticed positive correlations between the GBd and WHO grades (*p* = 0.005), PDCs-G (*p* < 0.0001), pN (*p* < 0.0001), pM (*p* = 0.019), TNM AJCC stage (*p* < 0.0001), LVI (*p* < 0.0001), PNI (*p* < 0.0001), tumor necrosis (*p* = 0.009), and the configuration of the tumor invasion front (*p* < 0.0001). Also, PDCs-G correlated positively with WHO grade (*p* < 0.0001), GBd (*p* < 0.0001), pN (*p* < 0.0001), TNM AJCC stage (*p* < 0.0001), LVI (*p* < 0.0001), PNI (*p* = 0.001), and the tumor border configuration (*p* < 0.0001).

Regarding the pT parameter, we observed positive correlations with tumor location, WHO grade, GBd, and PDCs (as shown above) but also with pN (*p* = 0.003), TNM AJCC stage (*p* < 0.0001), LVI (*p* < 0.0001), PNI (*p* = 0.002), and the tumor border configuration (*p* < 0.0001). The pN parameter correlated directly with WHO grade, GBd, PDCs, pT parameter, pM (*p* = 0.007), TNM AJCC stage (*p* < 0.0001), LVI (*p* < 0.0001), PNI (*p* < 0.0001), tumor necrosis (*p* = 0.023), and the configuration of the tumor invasion front (*p* < 0.0001). Distant metastases (pM1) associated with high-grade GBd (*p* = 0.019), pN+ (*p* = 0.007), advanced TNM AJCC stage (*p* < 0.0001), LVI+ (*p* = 0.001), PNI+ (*p* = 0.006), and the infiltrative type of the tumor invasion front (*p* = 0.047). In addition to the associations presented above, regarding the TNM AJCC stage, we also observed direct correlations that were statistically significant with LVI (*p* < 0.0001), PNI (*p* < 0.0001), tumor necrosis (*p* = 0.023), and the configuration of the tumor invasion front (*p* < 0.0001).

The LVI correlated positively and was statistically significant with the above-analyzed parameters but also with PNI (*p* < 0.0001), tumor necrosis (*p* = 0.023), and the model of the invasion front (*p* < 0.0001). So far, we have noticed essential associations of PNI with several parameters; moreover, PNI correlated positively with tumor necrosis (*p* = 0.032) and the type of tumor invasion front (*p* < 0.0001). Tumor ulceration did not correlate statistically with any of the analyzed parameters. Tumor necrosis correlated directly with some of the parameters assessed above; furthermore, we observed a direct relationship with the infiltrative type of the tumor invasion front (*p* = 0.040).

Regarding the tumor border configuration front, the infiltrative type of invasion presented a positive correlation with tumor localization in the right part of the colon (*p* = 0.036), a high WHO grade (*p* = 0.001), a high GBD grade (*p* < 0.0001), a high PDCs grade (*p* < 0.0001), the depth of tumor invasion into the intestinal wall pT3–pT4 (*p* < 0.0001), pN+ (*p* < 0.0001), pM1 (*p* = 0.047), advanced TNM AJCC stage (*p* < 0.0001), LVI+ (*p* < 0.0001), PNI+ (*p* < 0.0001), and tumor necrosis (*p* = 0.040). Tumor lymphocytic infiltrate did not associate significantly with the other parameters we analyzed.

## 4. Discussion

The identification of prognostic factors offering a perspective on the severity of the disease represents a significant concern in the field of oncological research nowadays. Although the parameters that determine the pathological stage of the disease—pTNM—are the most precise indicators of post-operative outcome, other clinical, histological and/or molecular features can influence the prognosis regardless of stage. Furthermore, molecular classifications are gaining more and more ground and have an essential role in cancers with various locations. Still, a series of histopathological characteristics that are much more affordable, easier to assess, and cheaper represent important elements to be investigated in CRC from the perspective of the identification of parameters that have an important role in the lymph nodal spread and the dissemination of this malignant tumor.

### 4.1. Patients’ Age and Sex and the Site of the Tumor

In this study, the patients’ age and sex did not correlate significantly with any other analyzed parameter. However, regarding the distribution of cases, we observed a higher incidence of CRC in patients over 65 years old. Also, the tumors from patients ≥65 years old were more frequently diagnosed in men, similar to data from the literature [2,26,27]. According to studies assessing the global incidence of cancer in older adults with CRC, new cases are expected to double by 2035 along with increasing life expectancy and the general aging of the population [26]. Young-onset CRC is more likely to be diagnosed at an advanced stage (pT3, pT4) with nodal or distant metastases, which indicates more advanced disease and poorer prognosis [27]. Still, most of these early-onset cases are sporadic. The risk of CRC is highest after the age of 40 years and begins to increase sharply between the ages of 50–55 years, doubling with each decade and continuing to grow exponentially [26]. In addition, these lesions’ underlying mechanisms and risk factors are not fully understood, warranting further research.

The localization of CRC correlated significantly with the extent of the tumor into the intestinal wall (pT1–T2/pT3–T4) and the configuration of the invasion front. Thus, tumors of the right colon were all pT3–pT4 and presented an infiltrative-type invasion model. In the specialty literature, the association between tumor localization and pT parameter has been described, with rectal tumors and those of the left colon being diagnosed in incipient stages of tumor extension due to early symptomatology and screening by colonoscopy [28]. According to one study, patients with proximal colon cancer had vague symptoms. They suffered from more comorbidities, so proximal colon cancer was diagnosed at a more advanced stage and was poorly differentiated compared to distal colon cancer [29]. Thus, it was hypothesized that the differences between proximal and distal colon cancers are probably due to different genetic mutations, lifestyle, and/or dietary habits. The risk of cancer in the proximal colon can be related to changes in bile metabolism (dyspepsia, gallstone disease) with the alteration of the gut microbiota [30,31]. However, data from the literature suggest that even patients with asymptomatic gallstones have an increased risk of CRC, especially for proximal colon cancer [31]. Moreover, some studies emphasize the risk of colorectal cancer post-cholecystectomy, which raises the hypothesis of the possible implications of certain particularities of the gut microbiota [32]. Also, tumor localization influences systemic treatment’s success and metastatic CRC evolution [33,34,35]. Therefore, tumors of the right colon have BRAF mutations more frequently; they present in a more advanced stage of the disease and are associated with a higher rate of post-surgical complications and a poorer prognosis than left colon tumors [34]. Also, the treatment is different; neoadjuvant radiotherapy is essential in treating rectal carcinomas [33,35].

### 4.2. Grading Systems

In our study, multivariate analyses were carried out only for the ADK NOS cases, leaving out the two cases of mucinous ADKs. We did not grade the mucinous ADKs with any of the three grading systems because, at present, there is no consensus about the definition of TB or PDCs in the presence of mucin.

In addition to the TNM stage, the histological grade (WHO grade) is an important prognostic factor in CRC. The tumor grade is consistently reported and recognized as one of the most critical parameters correlated with CRC aggressiveness [1]. Unfortunately, various CRC grading schemes have proven questionable in practice with significant inter-observer variability due to the lack of explicit and well-established diagnostic criteria [3,5]. Given these issues, the requirements for CRC grading needed to be refined. Currently, CRC grading is based on the percentage quantification of glandular structure formation [1,15]. In books of reference, the use of a binary grading system was recommended; if >50% of the tumor forms glands (G1 and G2 from the four-grade classification), CRC is considered to have low malignity; if <50% of the tumor presents tubular structures (G3 and G4 from the four-degree classification), CRC has a high malignity grade [1,15,36]. This revised classification is based on the similar evolution of patients with well and moderately differentiated ADKs [37]. We compared the newer systems of grading, GBd and PDCs, with the WHO grade. We observed a different distribution of CRC cases: By using the grading system based on the quantification of TB, most cases (49.3%) were classified as G3Bd (high-grade TB), while in the WHO classification, the majority of cases (79.7%) were considered G2. With the help of the grading system based on the assessment of PDCs, we observed a more uniform distribution of cases; most cases (44.9%) were classified as PDCs-G2 being followed closely by PDCs-G3 cases (39.1%). All three grading systems proved to have prognostic importance. Still, we noticed a more significant association of GBd and PDCs-G with the other prognostic parameters we analyzed compared to the WHO classification. GBd shows the strongest correlation with PDCs, pN, TNM AJCC stage, LVI, PNI, and the configuration of the tumor invasion front in all situations, the value of *p* being <0.0001. Unlike the WHO grade, multivariate analysis revealed a relationship between GBd and the pM parameter (*p* = 0.019) and tumor necrosis (*p* = 0.009).

The data we obtained support other studies that show a strong correlation between high-grade TB and other negative prognostic factors. Most research reports an adverse effect of the presence and high number of TB in correlation with LVI+, pN+, pM1, or the infiltrative type of tumor invasion front [25,38,39,40,41,42]. Some authors even consider TB an independent prognostic marker in CRC without lymph node metastasis [43]. TB is predictive for pN+ on resection specimens of early invasive (pT1 and pT2) rectal cancers, suggesting that it can be helpful as a prognostic indicator in patients with recurrence risk after local tumor excision [10,41]. Regarding therapy, the presence of TB on preoperative biopsies of CRCs would impose the application of neoadjuvant treatment [18]. According to some authors, in the case of malignant polyps and T1 tumors, TB represents a predictor of lymph node metastases, entailing segmentary resection with regional lymphadenectomy [18,42].

The PDCs parameter was not as thoroughly researched as TB, but similarly to data from the literature, our study also noted the importance of this parameter. Therefore, we observed that PDCs-G correlated more with pN, TNM AJCC stage, LVI, PNI, and the model of the invasion site than the WHO grade, in most instances, the values of *p* being <0.0001. After comparing the PDCs-G system with GBd, we noticed that by using PDCs-G, we obtained a statistically significant strong positive correlation with tumor extension into the intestinal wall (pT); conversely, with the help of GBd, we obtained more meaningful results for the pM parameter and tumor necrosis. Barresi et al. [11,21,44,45,46,47] consider that PDCs-G is easier to reproduce and interpret than GBd; also, they confirm and consolidate the idea that PDCs-G represents an adverse prognostic factor, which is more valuable compared to the classical histological grading system (WHO grade) and even to the TNM AJCC stage in patients with CRC. Also, PDCs-G proved to be a robust parameter for classifying pN+ risk in cases of CRC with incipient invasion of the intestinal wall (pT1) [48,49]. Moreover, it was demonstrated that high-grade PDCs in preoperative biopsies correlate with the infiltrative type of tumor invasion front, LVI+, and high-grade TB [47]. However, the maximum number of tumor cells forming the clusters, the optimum cut-offs for each PDC grade, and the quantification method for PDCs in particular histological types has not yet been established. Thus, although some studies demonstrate the value of PDCs in CRC, a consensus about the assessment and report of PDCs has not yet been reached.

### 4.3. TNM AJCC Stage of the Disease

Concerning the extension of the tumor into the intestinal wall (pT), we noted statistically significant positive associations with the other analyzed prognostic factors. Our results are similar to most studies showing that tumor extension into the intestinal wall and the TNM AJCC stage are the most important prognostic factors in patients with CRC [16,50]. The status of loco-regional lymph nodes (pN) represents an important prognostic factor in CRC; its assessment is part of the standard staging procedure. The presence of lymph node involvement (pN+) significantly reduces survival rates in patients with CRC according to the number of affected lymph nodes. The involvement of lymph nodes is considered “the second strong indicator of post-surgical evolution”, the first being the presence of distant metastases [22]. In our study, pN correlated positively with WHO grade, pT, pM, and tumor necrosis. Still, very significant correlations were observed between pN+ and G3Bd, PDCs-G3, advanced TNM AJCC stage, LVI+, PNI+, and the infiltrative type of the invasion site. Our results support the value of this parameter because in the literature, node status is recognized as one of the most important prognostic factors in CRC [39,49]. Distant metastases have a negative impact on patient survival, as shown in a meta-analysis where relative 5-year survival rates in patients with locally, regionally, and distantly advanced disease were 90.3%, 70.4%, and 12.5%, respectively [15]. We observed that the distant metastases correlated directly with G3Bd, pN+, PNI+, and the infiltrative configuration of the invasion front, a very significant relation being observed with LVI+ and advanced TNM AJCC stage. As established according to the AJCC/UICC system, the clinical–pathological stage is considered the most potent prognostic factor for patients with early-stage CRC. The situation is less clear for those with metastatic disease than for the intermediary stages of the disease [22] because patients with stage IIB and IIC tumors often show a lower survival rate than those with stage IIIA tumors [39]. In our study, the TNM AJCC stage showed a highly significant positive correlation with most analyzed features.

### 4.4. Lymphovascular and Perineural Invasion, Tumor Ulceration, and Necrosis

We observed significant associations (positive correlation) of LVI with the majority of the analyzed parameters, our results being in accordance with data from the literature [51]. In our study, we analyzed vascular invasion as a single parameter, putting together venous and lymphatic invasion because on usually stained sections, it is difficult to assess the type of vessels involved, especially with little vessels where endothelial cells are very hard to observe. However, some studies from the literature highlight the importance of differentiating venous from lymphatic invasion, as they have different prognostic implications [5,52,53,54]. In addition, we noticed important associations between PNI and the rest of the features with a negative impact on the prognostic of patients with CRC. Our results regarding PNI support the data from the literature where PNI is correlated with other negative prognostic markers, such as the presence of LVI, the high grade of malignity, or TB [52,55,56]. Also, we noted that tumor necrosis correlated with the parameters showing an adverse prognosis for patients with CRC, supporting the prognostic significance of tumor necrosis described in the specialty literature [52]. Pollheimer et al. consider that tumor necrosis is related to intra-/peritumor inflammation and microsatellite instability (MSI) [57].

### 4.5. Tumor Border Configuration and Tumor-Infiltrating Lymphocytes

Regarding the tumor border configuration, we observed that most cases (63.4%) presented an infiltrative configuration. This type of tumor invasion was associated with features that have a negative impact on the evolution of the disease, such as the localization of tumors in the right colon, an increased degree of malignity (G3–G4), the depth of invasion into the intestinal wall (pT3–pT4), an advanced TNM AJCC stage of the disease, pN+, pM+, LVI+, and PNI+, these results being in accordance with data from the specialty literature [58]. Some studies reported that the infiltrative type of invasion front was a significant predictor of lymph node metastases and recurrence in CRC [13,52].

Multiple studies reported that a high density of TILs correlates with a favorable prognosis in assessing different types of cancer, including CRC [14,23,59,60]. Nevertheless, the methods of TIL assessment are different from study to study, so there is no standardized and generally applicable methodology for evaluating TILs. On the other hand, it is not clear if the tumor invasion site is indeed the best area to assess TILs [61], but Kim et al. state that the evaluation of the invasion front reflects most accurately the characteristics of the neoplasm in patients with CRC and consider this the optimum site for TIL assessment [62]. In our study, according to the above-discussed approach and the model applied by Schwarz et al. [23], we evaluated TILs at the tumor invasion front, establishing a 5% cut-off to distinguish between the low TILs group (TIL−) and high TILs group (TILs+). We considered TILs to be mononucleate cells (lymphocytes, plasma cells) without taking into account granulocytes or macrophages. But, using this criterion, our results were different than those reported by other studies from the literature: TILs did not correlate significantly with the other prognostic factors we analyzed. Thus, it is possible that our assessment method was not the best choice. A possible explanation for the results we obtained could be, on the one hand, the selection of a single area to evaluate TILs instead of various regions throughout the tumor, reporting a mean density of TILs, and on the other hand, the use of two-scale classification (TIL−/TILs+) according to the 5% cut-off to the detriment of a type with more scales depending on the percentage of TILs. The assessment of TILs represents a possible future research direction. Still, standardized and generally accepted quantification methods and additional studies are necessary to validate the prognostic significance of TILs in CRC.

We reiterate the idea that quantification schemes are needed for these parameters that lead to uniformity and reproducibility in their assessment and reporting in daily clinical practice. Thus, the automated detection algorithms in TB, PDC, and TIL quantification and cumulative schemes for quantifying these parameters represent the next steps in further research.

### 4.6. Limitations of the Study

This is a retrospective study carried out in a single location on a relatively small number of patients. Another drawback of our study is the absence of clinical follow-up data that would favor the identification of possible correlations with short- and long-term survival outcomes.

## 5. Conclusions

Our study shows that the infiltrative type of tumor border configuration, a high TB grade, a high PDCs grade, and PNI present the highest correlation for lymphovascular invasion and lymph node dissemination in CRC. We emphasize that TB, PDCs, the tumor border configuration, and TILs, a series of parameters less studied but easy to evaluate on usually stained slides, are indicators that should be known. The pathologist should be familiar with their identification and reporting, which could allow the clinician to adjust the therapeutic management of patients by providing more aggressive treatments in the presence of some of these factors.

## Figures and Tables

**Table 1:** Clinical–pathological characteristics of the patients with CRC.

Parameters		*n* = 71	%
Sex	Male	44	62.0
	Female	27	38.0
Age of diagnosis	Median age 66.47 years		
	<65 years old	28	39.4
	≥65 years old	43	60.6
Site of tumor	Right colon	15	21.1
	Left colon	23	32.4
	Rectum	33	46.5
Depth of tumor invasion	pT1–pT2	11	15.5
	pT3–pT4	60	84.5
Lymph node status	pN−	37	52.1
	pN+	34	47.9
TNM AJCC stage of the disease	I	10	14.1
	II	28	39.4
	III	28	39.4
	IV	5	7.1
Lymphovascular invasion	LVI−	43	60.6
	LVI+	28	39.4
Perineural invasion	PNI−	48	67.6
	PNI+	23	32.4
Tumor ulceration	absent	10	14.1
	present	61	85.9
Tumor necrosis	absent	24	33.8
	present	47	66.2
Configuration of the tumor invasion front	pushing	26	36.6
	infiltrating	45	63.4
Tumor-infiltrating lymphocytes	TILs−	11	15.5
	TILs+	60	84.5

**Table 2:** The distribution of ADK NOS cases according to the histological differentiation degree using the three grading systems.

Parameters		*n* = 69	%
The usual grading system—WHO grade	G1	6	8.7
	G2	55	79.7
	G3–G4	8	11.6
The grading system based on TB quantification—GBd	G1Bd	16	23.2
	G2Bd	19	27.5
	G3Bd	34	49.3
The grading system based on PDCs quantification—PDCs-G	PDCs-G1	11	16.0
	PDCs-G2	31	44.9
	PDCs-G3	27	39.1

## Data Availability

Data generated or analyzed during this study are included in this published article and can be provided if needed or requested by the reviewer.

## Supplementary Materials

The following supporting information can be downloaded at: https://www.mdpi.com/article/10.3390/medicina59101761/s1, Table S1: The multivariate analyses for the cases of ADK NOS using the Pearson correlation coefficient (*n* = 69).
